# Comparative diffusion assay to assess efficacy of topical antimicrobial agents against *Pseudomonas aeruginosa *in burns care

**DOI:** 10.1186/1476-0711-10-27

**Published:** 2011-06-24

**Authors:** Fabien Aujoulat, Françoise Lebreton, Sara Romano, Milena Delage, Hélène Marchandin, Monique Brabet, Françoise Bricard, Sylvain Godreuil, Sylvie Parer, Estelle Jumas-Bilak

**Affiliations:** 1 Université Montpellier 1, UMR5119, Unité de Bactériologie, Faculté de Pharmacie, 15, Avenue Charles Flahault, BP 14491, 34093 Montpellier Cedex 5, France; 2 Centre Hospitalier Régional Universitaire de Montpellier, Service des Brûlés, Hôpital Lapeyronie, 371 Avenue du Doyen Gaston Giraud, 34295 Montpellier Cedex 5, France; 3 Centre Hospitalier Régional Universitaire de Montpellier, Hôpital La Colombière, Service d'Hygiène Hospitalière, 39 avenue Charles Flahault, 34295 Montpellier Cedex 5, France; 4 Centre Hospitalier Régional Universitaire de Montpellier, Laboratoire de Bactériologie, Hôpital Arnaud de Villeneuve,, 371 Avenue du Doyen Gaston Giraud, 34295 Montpellier Cedex 5, France

**Keywords:** *Pseudomonas aeruginosa*, burns, silver sulphadiazine, antiseptics, ERIC-PCR, diffusion assay

## Abstract

**Background:**

Severely burned patients may develop life-threatening nosocomial infections due to *Pseudomonas aeruginosa*, which can exhibit a high-level of resistance to antimicrobial drugs and has a propensity to cause nosocomial outbreaks. Antiseptic and topical antimicrobial compounds constitute major resources for burns care but in vitro testing of their activity is not performed in practice.

**Results:**

In our burn unit, a *P. aeruginosa *clone multiresistant to antibiotics colonized or infected 26 patients over a 2-year period. This resident clone was characterized by PCR based on ERIC sequences. We investigated the susceptibility of the resident clone to silver sulphadiazine and to the main topical antimicrobial agents currently used in the burn unit. We proposed an optimized diffusion assay used for comparative analysis of *P. aeruginosa *strains. The resident clone displayed lower susceptibility to silver sulphadiazine and cerium silver sulphadiazine than strains unrelated to the resident clone in the unit or unrelated to the burn unit.

**Conclusions:**

The diffusion assay developed herein detects differences in behaviour against antimicrobials between tested strains and a reference population. The method could be proposed for use in semi-routine practice of medical microbiology.

## Background

The current techniques of resuscitation, surgery and wound care have significantly improved the morbidity and the mortality of patients with burn wounds [1]. However, severely burned patients may still develop life-threatening nosocomial infections that remain a major challenge for burn teams [2]. The most frequent infections are wound infections, pneumonia, bloodstream and urinary tract infections [2,3]. Among the nosocomial pathogens, *Pseudomonas aeruginosa *from the patient's endogenous microflora and/or from the environment represents the most common isolated bacteria in many centres [2,4,5]. Infections with *P. aeruginosa *are particularly problematic since this bacterium exhibits inherent tolerance to several antimicrobial agents and can acquire additional resistance mechanisms turning inefficient all current antimicrobial drugs [6,7].

Antiseptic and topical antimicrobial compounds represent major resources in the therapeutic arsenal available for burns care. It is widely recognized that these agents have played a significant role in decreasing the overall fatality rate in burn units. Some of them such as povidone-iodine and chlorhexidine are used for antisepsis during wound care, therapeutic bathes, debridement and surgery. Others, prepared as ointment or unguent, provide antimicrobial effects associated to the 'mechanic' protection of the wound. For example, the use of cerium nitrate-silver sulphadiazine that forms a leather-like eschar on burn wounds allows surgical treatment to be delayed and enables sequential excision and grafting [8-10]. This wound treatment policy is supposed to improve the patient survival [8,11] and is increasingly used.

Resistance of *P. aeruginosa *to silver sulphadiazine has been previously documented [12]. In our unit, a *P. aeruginosa *clone multiresistant to antibiotics colonized or infected 26 patients over a 2-year period. Silver sulphadiazine susceptibility of this clone was questioned owing to long-time colonization or to refractory infections of the wounds. We comparatively investigated the susceptibility of the resident clone and unrelated *P. aeruginosa *strains to silver sulphadiazine and to the main topical antimicrobial agents currently used in the burn unit. For this purpose, we developed an optimized rapid method based on diffusion assay. This method appears suitable for semi-routine investigation of therapeutic failure or outbreak situation in burn unit and may be used to guide the choice of the most appropriate topical antimicrobial agent for patient's management.

## Material and Methods

### Patients, settings, samples and bacterial strains

The burn unit of the Academic Hospital of Montpellier is a French regional centre. The ward displays 6 intensive care unit rooms, 4 hospitalization rooms and 2 bathrooms. For microbiological analyses, serial samples are taken on admission to the intensive care unit or whenever required for clinical reasons. Extensive environmental samplings including water and surfaces are performed twice a year or whenever required during epidemic alerts. We retrospectively analysed strains of *P. aeruginosa *isolated from patients admitted to the burn unit from January 2005 to August 2007 as well as strains recovered from environment during the same period. All the culturable strains (n = 87) were included in the study. Thirteen strains of *P. aeruginosa *unrelated to the burn unit obtained from a collection of clinical strains were also included.

### Routine antimicrobial treatment of patients in the burn unit

Silver sulfadiazine (SSD), Flammazine^® ^(1% SSD) or Flammacerium^® ^(1% SSD + 2.2% cerium nitrate), is generally applied each two days. Mafenide acetate (Sulfamylon^®^) is occasionally used. Povidone iodine is used for wound rinsing during dressing and surgery. Patients are bathed every two days with water containing chlorhexidine. If a *P. aeruginosa *infection is suspected, the first-line treatment is piperacillin/tazobactam plus tobramycin.

### Microbiological analysis

The bacteria were isolated from clinical or environmental samples by standard microbiological procedures. *P. aeruginosa *was identified using Gram staining, positive oxidase reaction, production of pigments onto King A and King B media (Bio-Rad Laboratories) or API 20NE system (bioMérieux). The bacterial strains were stored at -80°C in a preservative medium (bacterial preservers, Technical Service Consultant Limited).

### Pulsed-field gel electrophoresis (PFGE) and ERIC-PCR typing

Pulsed-field gel electrophoresis (PFGE) after digestion by *Spe*I was performed as previously described [13]. The ERIC-PCR assay was performed as described by Mercier (1996) [14] with modifications. DNAs were extracted using the kit AquaPure Genomic DNA (Bio-Rad Laboratories) as recommended by the supplier. Enterobacterial repetitive intergenic consensus (ERIC) PCR conditions were validated using unrelated, closely related and identical isolates of *P. aeruginosa *(as determined by PFGE). ERIC-PCR was performed using 0.5 ml thin-walled PCR tubes in an Eppendorf MasterCycler^® ^thermal cycler. The reaction mix contained the following reagents: 2.5 U of Go*Taq *Flexi DNA polymerase (Promega) in appropriate buffer with 2 mM MgCl_2 _and 3.5% DMSO, 0.2 mM each deoxynucleoside triphosphate (Fermentas), 20 pmol of each primer (ERIC1 5'-CACTTAGGGGTCCTCGAATGTA-3', ERIC2 5'-AAGTAAGTGACTGGGGTGAGCG-3') and 50 ng of genomic DNA. The final reaction volume was adjusted to 50 μL. PCR amplification was performed with an initial denaturation step at 95°C for 3 min followed by 30 cycles of denaturation (90°C for 30 s), primers annealing (45°C for 1 min) and extension at 72°C for 4 min with a final extension at 72°C for 16 min. Amplicon (5 μL) was loaded with 6X loading buffer (50% saccharose, 0.1% bromophenol blue) into 1.5% agarose gel in 0.5X Tris-Borate-EDTA (TBE) buffer with 0.5 μg mL^-1 ^ethidium bromide. Electrophoresis was run at 80V for 3 h at room temperature. PFGE profiles were visually interpreted as follows: when two profiles were identical or differed by 3 or less than 3 DNA fragments the same letter was affected to the profiles. PFGE profiles differing by more than 3 bands were identified by different letters. The same nomenclature was used for ERIC profiles but numbers were used instead of letters.

### Antimicrobial susceptibility testing

Antibiotic susceptibility was tested by disk diffusion assay on Mueller-Hinton agar and interpreted according to the recommendations of the Antibiogram Committee of the French Microbiology Society (CA-SFM) (http://www.sfm-microbiologie.org/UserFiles/file/CASFM/casfm_2010.pdf). The antibiotics disks used (Bio-Rad, Marne-la-Coquette, France) were as follows: ticarcillin (75 μg), ticarcillin/clavulanic acid (75 μg/10 μg), piperacillin (75 μg), piperacillin/tazobactam (75 μg/10 μg), imipenem (10 μg), cefotaxime (30 μg), ceftazidime (30 μg), cefepime (30 μg), aztreonam (30 μg), gentamicin (10 UI), tobramycin (10 μg), nalidixic acid (30 μg), ciprofloxacin (5 μg), fosfomycine (50 μg). Colistin Minimal Inhibitory Concentration (MIC) was determined using Etest^® ^(AB BIODISK, Solna, Sweden) according CA-SFM protocol. Identification of resistance mechanisms was deduced from susceptibility testing by disk diffusion assay results according to Courvalin et al. [15].

Susceptibility to topical antimicrobial agents was tested by agar well diffusion (AWD) assay modified from Nathan et al. [16]. The surface of 5-mm-thick Mueller-Hinton agar plates was inoculated with a bacterial suspension visually adjusted to 0.5 Mc Farland (10^8 ^CFU/mL) and diluted 100 fold. Then, 8-mm diameter holes were made in agar plates with sterile die cutter and the wells were loaded with topical agents. The following topical agents were tested: 1% SSD (Flammazine^®^, Solvay), 1% SSD + cerium nitrate (SSDC) (Flammacerium^®^, Solvay), 5% mafenide acetate (Sulfamylon^®^), 10% povidone-iodine (Betadine Gel^®^) and 10% povidone-iodine (alcoholic solution) and chlorhexidine. Before loading, Betadine gel, SSD and SSDC were diluted at 1/2, 1/4 and 1/4 w/v respectively, in sterile distilled water to insure the reproducibility of pipetting. Aliquots of the commercialized products were weighted in microtubes in sterile conditions, conserved as recommended by the supplier and diluted extemporaneously. Then, wells were loaded with 150 μl of the diluted agent. This volume insured complete well loading with homogeneous contact between the agent and the well edge. The inhibition diameters were measured after 18 h of incubation at 37°C using the Antibiotic Zone Reader apparatus (Fisher Lilly).

### Statistical analysis

Analyses were performed either in duplicate or in triplicate in independent assays. For each strain and each antimicrobial agent, the mean inhibition diameter and the standard deviation were calculated. Differences in inhibition zone sizes between groups of strains were tested using Student's *t-*test. *P*< 0.05 was taken as statistically significant.

## Results

### Microbiology and antibiotics resistance of the *P. aeruginosa *isolates

A total of 100 *P. aeruginosa *isolates, including 67 clinical and 33 environmental isolates were available for retrospective analysis. Eighty-seven isolates were recovered from 26 hospitalized patients (n = 55) or from environment (n = 32) in the burn unit. Thirteen additional isolates corresponding to 12 clinical samples and to 1 environmental sample formed a collection of hospital isolates epidemiologically unrelated to those of the burn unit. Origin of the isolates was given in tables 1, 2 and 3.

**Table 1:** Characteristics of the 42 *P. aeruginosa *strains from the burn unit with MDR phenotypes

Strain	Date of isolation	Patient and/or Origin		P	E	ATB	SSD	SSDC	BetG	BetL	Sulf	Chlor
PAB03	02-2005	4	Burn wound	A	1	MDR1	15.8	14.4	17.8	18.8	43.0	19.9
PAB07	03-2005	3	Burn wound	A	1	MDR1	9.1	8.0	19.9	21.0	43.0	21.0
PAB08	03-2005	4	Burn wound	A	1	MDR1	13.9	13.0	18.7	17.6	42.0	18.6
PAB13	04-2005	1	Burn wound	A	1	MDR1	11.4	11.2	20.8	19.0	46.0	20.6
PAB14	04-2005	1	Burn wound	A	1	MDR1	12.8	11.2	21.8	18.7	47.5	20.5
PAB15	04-2005	4	Burn wound	A	1	MDR2	16.4	14.8	19.2	18.5	45.0	19.4
PAB18	05-2005	1	Burn wound	A	1	MDR1	12.0	10.2	23.0	18.6	48.0	20.4
PAB20	07-2005	8	Respiratory tract	A	1	MDR1	15.4	15.0	17.7	18.4	45.0	19.1
PAB21	07-2005	8	Respiratory tract	A	1	MDR1	14.4	13.5	17.8	17.0	44.0	20.0
PAB22	07-2005	2	Urine	A	1	MDR1	15.2	13.4	21.0	19.8	46.0	21.6
PAB23	07-2005	2	Urine	A	1	MDR1	16.5	15.1	21.8	19.1	48.0	20.5
PAB25	07-2005	8	Respiratory	A	1	MDR2	15.2	15.0	22.2	18.0	47.0	20.6
PAB26	07-2005	8	Respiratory	A	1	MDR2	14.8	15.2	18.6	18,0	49.0	19.8
PAB32	11-2006	10	Burn wound	ND	1	MDR2	14.9	14.8	19.5	18.4	44.6	19.7
PAB33	01-2007	7	Burn wound	ND	1	MDR1	11.4	11.2	21.8	19.4	43.0	19.0
PAB34	02-2007	6	Burn wound	ND	1	MDR2	11.8	12.2	20.2	16,0	44.0	17.8
PAB35	02-2007	11	Burn wound	ND	1	MDR2	11.0	11.2	23.2	17.0	46.0	20.0
PAB37	03-2007	5	Burn wound	ND	1	MDR1	12.0	12.6	22.6	18.0	45.0	20.4
PAB39	01-2007	9	Burn wound	ND	1	MDR1	14.9	13.8	18.9	19.7	44.0	20.1
PAB42	01-2007	6	Burn wound	ND	1	MDR2	10.8	11.8	18.6	17.6	48.0	17.8
PAB43	02-2007	6	Blood	ND	1	MDR2	12.2	11.5	18.1	19.8	46.5	20.4
PAB44	02-2007	11	Urine	ND	1	MDR2	12.0	11.0	17.6	15.8	45.0	17.6
PAB45	02-2007	6	Respiratory	ND	1	MDR1	10.8	11.8	18.2	17.6	46.0	18.6
PAB46	03-2007	6	Burn wound	ND	1	MDR1	10.6	11.2	17.4	18.4	44.0	20.2
PAB47	03-2007	9	Burn wound	ND	1	MDR1	15.2	15.2	19.6	19.6	45.0	21.2
PAB48	03-2007	5	Burn wound	ND	1	MDR1	11.7	11.8	19.7	17.8	44.6	18.8
PAB50	04-2007	5	Respiratory tract	ND	1	MDR1	11.6	11.2	20.4	17.3	45.0	18.6
PAB29	10-2005	12	Burn wound	A	1	MDR2	11.2	12.2	21.2	16.8	44.0	19.4
PABH01	10-2006	Trap		ND	1	MDR2	14.8	14.0	20.0	18.4	47.0	19.6
PABH02	11-2006	Endoscoscope		ND	1	MDR2	14.4	14.4	19.2	17.4	46.0	19.8
PABH03	12-2006	Endoscoscope		ND	1	MDR2	14.8	14.2	18.0	17.2	46.0	20.2
PABH04	12-2006	Endoscoscope		ND	1	MDR2	14.8	14.8	19.4	18.3	49.0	18.7
PABH05	12-2006	Faucet		ND	1	MDR2	15.2	14.6	21.0	18.6	45.0	21.2
PABH06	12-2006	Endoscoscope		ND	1	MDR2	16.2	15.6	23.6	19.6	48.0	20.6
PABH07	12-2006	Endoscoscope		ND	1	MDR2	14.0	14.4	18.0	19.0	43.0	19.2
PABH08	NP	Trap		A	1	MDR1	15.4	15.2	22.4	19.0	47.0	22.0
PABH11	NP	Trap		A	1	MDR2	11.2	12.0	21.0	15.6	42.0	19.6
PABH15	04-2007	Trap		ND	1	MDR2	16.4	15.6	19.8	17.4	43.0	19.8
PABH16	04-2007	Trap		ND	1	MDR2	17.8	16.6	21.6	18.6	47.0	20.0
PABH17	NP	Mattress		A	1	MDR1	12.2	12.6	21.0	18.9	37.5	19.4
PABH23	01-2007	Trap		ND	1	MDR1	15.2	15.2	20.4	19.2	45.0	21.0
PABH29	01-2007	Basin washing-machine		ND	1	MDR2	15.0	15.2	21.4	18.0	42.0	20.0

(P) for PFGE profile; (E) for ERIC-PCR type; (ATB) for antibiotics susceptibility phenotype and AWD inhibition diameters in mm for (SSD): silver sulphadiazine, (SSDC): cerium nitrate-silver sulphadiazine, (BetG): povidone iodine gel, (BetL):povidone iodine solution, (Sulf): mafenide (Sulfamylon^®^), (Chlor): chlorhexidine. ND not determined.

**Table 2:** Characteristics of 45 *P. aeruginosa *strains from the burn unit with non-MDR phenotypes^1^

Strain	Date of isolation	Patient and/or Origin		P	E	SSD	SSDC	BetG	BetL	Sulf	Chlor
PAB01	01-2005	13	Burn wound	G	7	24.7	24.3	24.3	19.3	46.5	17.9
PAB02	01-2005	13	Burn wound	G	7	25.0	24.4	21.6	18.6	47.0	16.4
PAB04	02-2005	24	Burn wound	H	39	23.0	22.0	20.4	17.4	51.0	14.6
PAB05	03-2005	16	Burn wound	E	2	26.0	23.8	18.7	18.2	48.0	16.1
PAB06	03-2005	16	Burn wound	E	2	25.6	25.2	21.1	17.4	44.6	19.6
PAB09	03-2005	26	Urine	F	36	25.6	25.6	22.7	18.2	47.0	18.0
PAB10	03-2005	26	Respiratory	F	36	24.0	23.6	21.6	17.2	42.0	17.2
PAB11	03-2005	26	Respiratory	F	36	24.0	24.6	20.8	17.0	44.0	15.0
PAB12	04-2005	16	Blood	E	2	25.0	21.6	21.2	17.6	45.0	20.4
PAB16	04-2005	16	Urine	E	2	26.8	25.8	23.7	17.5	44.5	21.2
PAB17	04-2005	16	Urine	E	2	27.6	26.0	22.6	18.0	48.0	22.0
PAB19	06-2005	20	Burn wound	I	14	22.6	22.8	18.9	19.9	45.0	24.1
PAB24	09-2005	23	Burn wound	J	37	25.0	23.6	21.2	18.6	46.0	20.6
PAB27	08-2005	15	Burn wound	ND	6	16.0	14.6	21.6	19.6	47.0	20.4
PAB28	08-2005	15	Burn wound	ND	6	26.1	27.3	21.7	17.1	43.6	18.9
PAB38	01-2007	19	Urine	ND	11	16.0	15.4	19.2	18.2	47.0	20.6
PAB40	01-2007	19	Burn wound	ND	35	20.9	21.8	19.4	17.4	48.5	19.2
PAB41	01-2007	19	Burn wound	ND	11	15.2	15.4	17.2	18.0	46.0	20.4
PAB49	04-2007	18	Burn wound	ND	10	21.8	20.3	20.3	18.6	45.3	19.6
PAB52	04-2007	21	Respiratory	ND	15	24.7	23.6	18.9	17.5	45.6	15.0
PAB53	05-2007	25	Urine	ND	16	25.6	26.0	18.7	17.0	37.0	18.5
PAB54	05-2007	14	Burn wound	ND	38	27.3	29.0	22.6	20.3	45.6	17.8
PAB55	05-2007	18	Burn wound	ND	10	16.0	14.8	20.2	17.0	43.0	20.2
PAB61	06-2007	22	Burn wound	ND	17	24.0	23.6	19.2	18.8	48.0	21.0
PAB63	07-2007	14	Urine	ND	18	22.4	22.6	19.8	18.0	46.0	18.6
PAB66	08-2007	17	Burn wound	ND	9	25.8	23.8	20.0	16.2	44.0	17.6
PAB67	08-2007	17	Blood	ND	9	25.0	24.2	20.0	18.6	45.0	17.6
PABH09	NA	Trap		K	3	20.0	18.6	17.2	16.6	40.0	15.2
PABH10	NA	Trap		ND	19	19.6	20.0	17.0	17.4	44.0	24.2
PABH12	NA	Basin washing-machine		ND	4	23.4	22.6	21.0	18.2	42.0	17.0
PABH13	NA	Basin washing-machine		ND	4	23.0	23.2	22.4	18.2	41.0	13.0
PABH14	NA	Water		K	3	24.4	26.2	21.6	17.0	42.0	16.0
PABH19	10-2006	NA		ND	21	25.1	23.9	21.1	17.6	42.0	18.6
PABH20	10-2006	Basin washing-machine		ND	22	26.2	25.4	23.3	20.4	44.0	18.8
PABH21	10-2006	Water		ND	4'	28.9	27.6	21.2	16.8	46.0	18.8
PABH22	10-2006	Shower		ND	34	27.6	30.5	22.9	18.9	45.5	15.8
PABH24	01-2007	Trap		ND	3'	22.4	21.2	22.2	19.8	42.0	17.6
PABH25	01-2007	Mattress		D	34	26.2	26.2	18.0	17.1	45.0	16.7
PABH26	01-2007	Table		ND	34	26.6	25.8	22.6	16.0	45.0	19.2
PABH27	01-2007	NA		ND	34	26.4	27.2	23.8	15.8	44.0	17.8
PABH28	01-2007	Trap		ND	3	27.0	27.8	23.4	19.0	43.0	17.8
PABH30	01-2007	Basin washing-machine		ND	23	26.2	25.0	24.4	19.6	43.0	19.0
PABH31	05-2007	Infusion support		ND	24	26.8	25.6	19.8	19.6	43.0	16.0
PABH33	05-2007	Faucet filter		ND	25	21.2	18.6	17.6	18.6	43.0	20.6
PABH34	01-2007	NA		ND	26	23.0	22.8	23.2	18.0	44.0	17.8

See Table 1 for legend. NA: data not available.

^1^Antibiotics susceptibility phenotypes were highly diverse among the non-MDR isolates

**Table 3:** Characteristics of the 13 *P. aeruginosa *strains unrelated to the burn unit

Strain	Date of isolation	Origin	P	E	SSD	SSDC	BetG	BetL	Sulf	Chlor
PAE 1	1992	Eye		27	26.1	24.7	18.0	18.2	46.5	21.5
PAE 3	1985	Orthopedic wound		35	25.0	24.6	19.2	15.8	46.0	17.9
PAE 7	1985	Eye		37	27.2	26.2	20.2	20.6	48.0	17.6
PAE 15	1986	Orthopedic wound		29	28.2	27.4	19.4	19.0	47.0	14.6
PAE 16	1986	Orthopedic wound		28	27.8	27.8	20.4	20.4	46.0	17.6
PAE 36	1985	NA		36	25.8	29.0	18.8	17.8	46.0	15.4
PAE 37	1985	NA		30	27.0	29.0	24.2	20.0	44.0	16.2
PAE 40	1985	NA		31	29.0	30.2	21.4	17.4	47.0	21.2
PAE 41	1997	Eye		32	29.0	31.4	20.4	18.0	42.0	18.6
PAE 30	2006	Respiratory tract		8	21.8	20.2	21.2	19.0	46.0	20.4
PAE 31	2006	Drain		8	20.4	20.6	22.0	17.2	48.0	20.6
PAE 70	2007	Drain		20	20.7	20.8	21.3	17.8	44.5	21.4
PAE 32	2007	Water		33	24.2	25.8	18.8	17.4	44.0	23.6

See Table 1 and 2 for legend. All the strains showed non-MDR phenotype^1^.

^1 ^Antibiotics susceptibility phenotypes were highly diverse among the non-MDR isolates

Forty-two isolates of the burns unit displayed antimicrobial susceptibility profiles with resistance to about all commercially available antibiotics tested. Among them, eighteen clinical and 3 environmental strains resisted to all beta-lactams including imipenem, to aminoglycosides, to ciprofloxacine and to fosfomycin. This multi-drug resistance pattern will be named MDR1 (Table 1). Closely related pattern, named MDR2, grouped 10 clinical and 11 environmental strains resistant (R) to all antibiotics tested but susceptible (S) to fosfomycin (Table 1). For the strains with MDR1 and MDR2 phenotype, the colistin MIC value was from 4 to 8 μg/mL. No MDR1 or MDR2 phenotype was observed in the unrelated strains collection. Other isolates from the burns unit or not (Tables 2 and 3) showed various resistance patterns. Regarding beta-lactams, we observed wild type phenotype, cephalosporinase overexpression, penicillinase production, oxacillinase production, efflux pumps overexpression, porin D2 impermeability or complex phenotypes associating several of the previous resistance mechanisms. The strains displayed various behaviours against fluoroquinolones, aminoglycosides and fosfomycin.

### Molecular typing of *P. aeruginosa*

**Figure 1:**
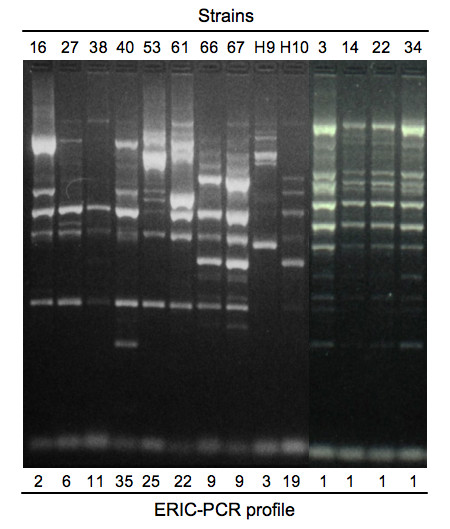
**Selected ERIC-PCR profiles**. The strains analyzed were PAB16, PAB27, PAB28, PAB40, PAB53, PAB61, PAB66, PAB67, PABH9 and PABH10 and were indicated at the top of the gel. ERIC-PCR profiles were indicated at the bottom of the gel.

We analysed all the bacterial population (n = 100) by ERIC-PCR and a comparison to PFGE was performed for about one third of strains (n = 33). Interpretable ERIC-PCR pattern was obtained for all isolates. A gel representative of the ERIC-PCR patterns is shown in Figure 1. The strains were distributed in 36 distinct ERIC-PCR profiles (Tables 1, 2 and 3). PFGE confirmed the ERIC-PCR-based clustering (Table 1 and 2) for the 33 strains analysed by both methods, thereby validating the PCR-based results. The 55 clinical strains and the 32 environmental strains displayed 17 and 11 different ERIC-PCR profiles, respectively. The strains unrelated to the burn unit were more diverse since 12 different profiles were observed for the 13 strains. A main ERIC-PCR profile type, named ERIC1, was observed for 42 isolates corresponding to 28 clinical strains isolated from 13 different patients and 14 environmental isolates from the burns unit (Table 1). The ERIC1 profile was never found in strains unrelated to the burn unit. The strains with ERIC1 profile have been isolated from February 2005 to April 2007. All these isolates were multi-resistant to antibiotics and displayed the resistance pattern MDR1 or MDR2. The 45 other isolates from the burns unit displayed 23 other different ERIC-PCR patterns and none of them were of MDR1 or MDR2 phenotype (Table 2 and 3). Out of the ERIC1-type group, the strains sharing the same ERIC-PCR profile were isolated from the same burn patient and the same ERIC-PCR profiles were not shared between clinical and environmental strains in the burn unit. The strains unrelated to the burn unit displayed ERIC-PCR patterns that were not observed in the burn unit. Again, in this group, the same pattern was obtained only for strains isolated from the same patients. Finally, genomotyping showed that MDR1 and MDR2-type strains are clonal and that this clone persisted over a 2-years period in the burn unit.

### Optimization of the agar well diffusion (AWD) assay for topical agents

The wells were filled with agents in their commercial forms except for semi-solid forms, which need to be diluted to insure the reproducibility of the wells pouring. A range of binary dilutions from pure to 1/8 was tested on 5 selected bacterial strains. The resulting inhibition diameters did not vary significantly for Flammazine^® ^(from 17 to 15 mm) and for Flammacerium^® ^(from 20 to 18 mm). For Betadine^® ^gel, the range of inhibition zone was wider, from 27 to 20 mm when the dilution increase. The absence of defined cut-off values for inhibition diameter in AWD assays imposed a comparative approach for the results interpretation. Therefore, attention should be given to the reproducibility of the method rather than to the absolute diameter measuring. In all cases, the edges of the inhibition zones were more regular and clear when the agents were diluted. We chose for each agent the lowest dilution insuring easy and reproducible pipetting and wells pouring: 1/2, 1/4 and 1/4 w/v for Betadine gel^®^, SSD and SSDC respectively.

The AWD method has also been improved by testing different bacterial inoculums. Bacterial charge affected significantly the diameter of inhibition (data not shown). This was particularly obvious for the Sulfamylon^® ^diameter which was large (> 40 mm) and not clearly delimited with micro-colonies growing in the border of the main diameter. Inoculation of the plates with 10^6 ^CFU gave the more interpretable results. With this inoculum, clear-cut and easy to read diameters were obtained for all topical agents. Particular care should be taken for the preparation of the inoculum in order to insure reproducibility of the AWD tests. This optimized protocol is compatible with a semi-routine practice of medical microbiology since about 10 strains could be analysed over a 1-hour period of bench manipulation, including dilution of commercialized agents aliquots.

### Activity of the topical antimicrobial compounds

Since the method AWD was not standardized and reference strains were unavailable for antimicrobial assays on topical agent, we undertook AWD assays with comparison of results at the population level.

First, the mean inhibition diameter for each topical agent was compared with the results of Pirnay et al. [12], at the whole population level. Mean diameter for SSD, SSDC, chlorhexidine, iodine-povidone and Sulfamylon^® ^were respectively 19.7 mm, 19.4 mm, 19.3 mm and 44.9 mm in our study and 20.2 mm, 21 mm, 19.1 mm and > 30 mm in the study of Pirnay et al. [12]. The similarity of the mean diameters in two population of *P. aeruginosa *isolated in burns units gave arguments to validate our AWD approach.

**Figure 2:**
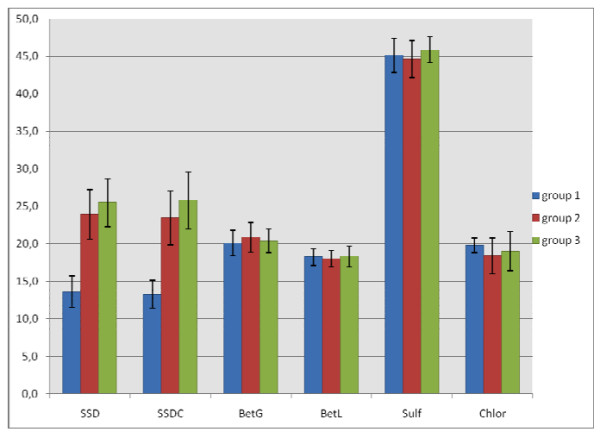
**Repartition of the AWD diameter according topical antimicrobial agents and group of strains**. Abbreviations of topical agents names as defined for table 1. Group of strains as defined in the text. Inhibition zone diameters in mm; Bar, standard deviation.

Secondly, we undertook a comparative AWD assay between isolates belonging to the MDR1/2-ERIC1 clone (group 1; n = 42) and unrelated *P. aeruginosa *strains from the burns unit (group 2; n = 45) or from elsewhere (group 3; n = 13). The results of the comparative AWD tests were presented in tables 1, 2 and 3 and summarized in Figure 2. The isolates belonging to group 1 displayed significant decrease of SSD and SSDC inhibition diameters comparatively to group 2 and 3 (P < 0.001) (Figure 2). For chlorhexidine, iodine-povidone and Sulfamylon^® ^no significant differences in inhibition diameters were observed among the 3 groups (P > 0.05) (Figure 2). In spite of a selective pressure of topical agents similar to group 1, most of the group 2 strains displayed inhibition diameters corresponding to those observed in the group 3 for all agents tested. However, 4 strains affiliated to group 2 (PAB27, PAB38, PAB41, PAB55) showed inhibition diameters similar to strains of group 1. The strains PAB38 and PAB41 isolated from the same patient displayed the ERIC-PCR 11 profile and a wild type phenotype regarding the resistance to antibiotics. This indicated that the low susceptibility to SSD and SSDC was not obligatory associated with multi-resistance to other antimicrobial agents. The isolate PAB55, belonging to the ERIC-PCR profile 10, also showed limited diameter around SSD and SSDC wells and a wild phenotype regarding antibiotics. In the same ERIC group, the strain PAB49 was isolated from the same patient one month before. This isolate did not display reduced susceptibility to topical agents but displayed a phenotype of penicillinase producer. Other situation, the strains PAB27 and PAB28 sharing the genomotype ERIC6 were isolated on the same day from burn wounds of the patient 15. The 2 strains presented the same wild antibiotypes but PAB27 only showed limited diameter around SSD and SSDC. This suggested that in a same genomotype the resistance patterns to antibiotics and/or topical antimicrobial agent could vary rapidly. Another hypothesis was the co-existence of mixed populations harbouring diverse phenotypes against antimicrobial agents.

## Discussion

We proved by PFGE and ERIC-PCR that 42 strains isolated from the environment and from the patients of the burn unit over a 2-year period belonged to the same clone. They displayed the multi-drug resistant phenotypes MDR1/2. Comparison of PFGE to recent sequence-based typing methods such as Multi-Locus Sequence Typing [17], Single Nucleotide Polymorphism [18], Variable Number of Tandem Repeats [19] showed that PFGE remained the most discriminative method and is still considered as the "gold standard" for molecular epidemiology of *P. aeruginosa *[20]. This suggested that genetic changes in *P. aeruginosa *occurred by large rearrangements rather than by point mutations in housekeeping genes. Other genomotyping methods that also explored genomic rearrangements, such as rep-PCR, were slightly less discriminative than PFGE but have proved their efficiency for typing *P. aeruginosa *isolates in endemic or epidemic settings [21,22]. PCR-based approaches have the great advantage to be rapid, easy and cost-effective methods comparatively to PFGE [20].

The MDR1/2-ERIC1 clone could be considered as endemic and prevalent in the burns unit. Such resident multi-drug resistant strains have been previously reported [12,23]. In one case, the endemic strain evolved gradually from a moderate resistant to a multi-drug resistant phenotype [12]. Here, the resistant phenotype MDR1/2 appeared stably installed. However, we are not able to retrospectively perform the detection of ERIC1 genotype eventually associated with other antibiotic resistance patterns before 2005. A long-time persistent bacterial clone in a burn unit is submitted to the selective pressure imposed by the general use of topical antimicrobial agents. Owing to clinical evidence of low efficiency of local treatment upon wounds colonized with MDR1/2 clone, we undertook the *in vitro *testing of these strains regarding topical agents. As previously reported in a burn unit [12], we observed a decrease of susceptibility to SSD and SSDC of the isolates belonging to MDR1/2-ERIC1 clone. We also observed for two isolates that the low susceptibility to SSD and SSDC was not obligatory associated with the genomotype ERIC1 and/or with multi-resistance against antibiotics. In a recent study based on AWD assays, authors showed that 88% of non multi-drug resistant strains of the genera *Acinetobacter, Pseudomonas, Klebsiella, Staphylococcus *and *Enterococcus *were fully susceptible to topical agents compared to 80% of multi-drug resistant strains of the same genera [24]. We described for two pairs of strains isolated from the same patient (PAB49/55 and PAB27/28) rapid variation of their behaviour against antibiotics and/or topical agents. These variations could be explained by the co-existence of diverse sub-populations inside a same genomotype. Independent to their mechanism, the variations led to rapid adaptation in response to new selective pressures and probably according to the lowest energetic cost for the strain [25].

In spite of its use for 40 years ago, silver-sulphadiazine remains widely used today for topical antimicrobial treatment of burns [1]. Considered that its antiseptic capabilities were not sufficient in all cases, a second mineral nitrate, cerium nitrate, has been added to SSD in the SSDC unguent. SSDC was shown to reduced infections as observed for SSD but also led to significant increase in survival rate of patients with a large percentage of total body surface area burned, even in presence of sepsis. According to the burn centre, one observed 59% [9] and 39% [26] higher than expected survival rate when SSD and cerium nitrate were used in combination. It was generally recognized that cerium did not significantly enhanced the antimicrobial effect of SSD [27]. We confirmed here that the behaviour of *P. aeruginosa *against SSD and SSDC was similar *in vitro*. Therefore, the reduction in mortality rate might be attributed to the mechanic properties of SSDC that forms a leather-like protective and soft crust instead of the moist macerated eschar produced with SSD cream. SSD and SSDC were the more frequently used topical treatments in our unit since more than 95% of the patients entering the unit after thermal injuries were treated with Flammazine^® ^(SSD) and/or Flammacerium^® ^(SSDC). For patients with large burned surface, SSDC was used before excision and graft. The central place of SSD and SSDC in burn therapy, as well as the description of bacterial strains with reduced susceptibility to these agents urge the availability of efficient methods for their *in vitro *susceptibility testing.

Most topical antimicrobial efficacy studies in thermally injured patients are established in vivo in the Walker-Mason rat burn model in which a bacterial strain is applied to a 20% scald burn with or without the tested topical agent [28]. This method could not be performed routinely. *In vitro*, diffusion methods for topical agents were proposed 30 years ago but did not encountered the success of the Kirby-Bauer method applied to antibiotics. However, most recent reports referring to diffusion methods for testing topical agents underlined that these methods were the simplest and the most reproducible [12,24,29]. The use of disks as support of the tested agents was not possible for all agents. Particularly for creams, unguents or gels such as SSD, SSDC or Betadine Gel^® ^well loading was obligatory. For some authors, the correlation between *in vitro* testing and the clinical efficiency of topical agents is supposed to be low particularly because the *in vitro* assays explored bacteria in planktonic phenotype whereas the wounds are more likely to be colonized by bacteria with biofilm phenotype [30]. Considering this restriction, AWD assays with bacteria inoculated onto agar plates could present some advantages in comparison to methods using liquid broth. From a more general point of view, *in vitro* evaluation of bacterial susceptibility to topical agents and antiseptics suffer from the lack of standardization and defined cut-off values helping therapeutic decision. There are no specific tests for evaluating the efficacy of topical antimicrobials, including Minimal Inhibitory Concentration (MIC) determination, which have been standardized and approved by any oversight comity. Then, their use for the a priori prediction of clinical efficiency, as done with antibiogram, should not be currently recommended. Considering these limitations, we proposed (1) to undertake topical AWD assays on *P. aeruginosa *isolates owing to the preliminary evidence of low efficiency of local treatments, (2) to perform comparative analysis between the isolates of interest and unrelated *P. aeruginosa *strains. The inhibition diameters determined on a large reference population could be determined once and then used as a reference database. In semi-routine conditions, i.e. in response to a particular clinical situation, each clinical isolate should be tested in comparison with two strains of the reference population as controls. Moreover, the detection of MDR strains and/or endemic resident clone should lead to the determination of susceptibility to topical agents although these situations should not be strictly considered as pre-requisites before undertaking AWD assays. *In vitro* study of the mechanism of topical agent resistance should also be explored.

In our experience, the epidemic clone led to long-time wounds colonization and to refractory infections, suggesting the clinical significance of AWD assays on topical agents. Indeed, such long-time colonization and/or infection of burn wounds could be due to a less efficiency of SSD and SSDC. Unfortunately, precise clinical indicators could not be reported in this retrospective study. Further studies are required to conclude about the clinical significance of optimized comparative AWD assay on topical antimicrobial agents and about the benefice for the patients when this assay is performed in routine practice.

## Competing interests

The authors declare that they have no competing interests.

## Authors' contributions

FA performed molecular experiments, coordinated AWD tests and analyzed data, FL is the principal clinical investigator and is involved in the manuscript drafting, SR participated to the study design and data acquisition, MD performed and interpreted AWD tests, HM interpreted results and revised the manuscript, MB is a clinical investigator involved in the critical analyse of results, FB design and performed environmental investigations, SG performed and interpreted antibiotics testing, SP designed the study and helped to draft the manuscript and EJB conceived and coordinated the study and write the manuscript. All authors read and approved the final manuscript.

## Aknowledgments

We are grateful to Jean-Luc Jeannot for his help in topical agents manipulation. This study was partially supported by the association ADEREMPHA, Sauzet, France.
